# Role of S1P‐ and Rho‐kinase signalling in age‐related myogenic tone deficiency in murine resistance arteries

**DOI:** 10.1113/EP093296

**Published:** 2025-11-17

**Authors:** Gry Freja Skovsted, Alex Aupetit, Karl Björling, Kristian Agmund Haanes, Susanne Syberg, Niklas Rye Jørgensen, Blanca I. Aldana, Hirotsugu Tsuchimochi, Mark T. Waddingham, Kristine Freude, James Todd Pearson, Lars Jørn Jensen

**Affiliations:** ^1^ Department of Veterinary and Animal Sciences, Faculty of Health and Medical Science University of Copenhagen Frederiksberg Denmark; ^2^ Translational Research Centre Copenhagen University Hospital Rigshospitalet Glostrup Denmark; ^3^ Department of Biology University of Copenhagen Frederiksberg Denmark; ^4^ Danish Headache Center, Department of Neurology Copenhagen University Hospital Rigshospitalet Glostrup Denmark; ^5^ Department of Clinical Biochemistry Copenhagen University Hospital Rigshospitalet Copenhagen Denmark; ^6^ Department of Drug Design and Pharmacology, Faculty of Health and Medical Science University of Copenhagen Frederiksberg Denmark; ^7^ Department of Cardiac Physiology National Cerebral and Cardiovascular Center Research Institute Suita Osaka Japan

**Keywords:** ageing, G‐protein coupled receptors, myogenic tone, resistance arteries, Rho‐kinase, S1P‐signalling

## Abstract

Ageing is a risk factor for cardiovascular and neurodegenerative diseases. The myogenic response in resistance arteries is responsible for basal (myogenic) tone and blood flow autoregulation. G‐protein‐coupled receptors and G_12_/RhoA/Rho kinase are implicated in myogenic tone (MT), and we aimed to clarify their role in pressure sensing and ageing. We studied MT in third‐order mesenteric arteries (MA) *ex vivo* and first–fourth order cerebral arteries (CA) in vivo in young versus middle‐aged male mice. Inhibition of α_1_‐, AT_1_‐, ET_A_‐ and TP‐receptors and thromboxane synthase did not affect MT in MA from young mice. The P2Y‐receptor blocker suramin inhibited MT, whereas PPADS and apyrase did not. MT in intact or endothelium‐denuded MAs was not affected by the knockout of P2Y_6_‐receptor (P2Y_6_‐R). qPCR showed upregulation of P2Y_2_‐R in P2Y_6_‐deficient arteries. MT was not affected in P2Y_2_‐R knock‐out mice. The sphingosine‐kinase (SK) blocker SKI‐II inhibited MT in young mice, and the sphingosine 1‐phosphate receptor 2 (S1P_2_‐R) blocker JTE‐013 inhibited MT in young and middle‐aged mice. MT was impaired in middle‐aged mice. Furthermore, MT was reduced in young mice carrying familial Alzheimer's disease mutations (5xFAD), and JTE‐013 abolished MT in 5xFAD mice and their wild‐type littermates. JTE‐013 did not affect calcium signalling in cultured human coronary artery smooth muscle cells. High‐resolution microangiography confirmed that infusion of JTE‐013 or KD025 (a Rho‐kinase 2 inhibitor) preferentially dilated small (distal) CAs, and infusion of nifedipine (an L‐type channel inhibitor) dilated all CAs in all mice, independent of age. SK and S1P_2_‐R are crucially involved in pressure sensing in MT. RhoA/Rho‐kinase signalling might be involved in age‐related MT deficiency.

## Abstract (highlights)

- **What is the central question of this study?**
  What are the relative importance and age‐dependent roles of G‐protein‐coupled receptors in mesenteric and cerebral arteries in mice?
- **What is the main finding and its importance?**
  Data show no involvement of α_1_‐, AT_1_‐, ET_A_‐, TP‐, or P2Y_2_/P2Y_4_/P2Y_6_‐receptors, but suggest a S1P_2_‐receptor/Rho‐kinase signalling cascade in mesenteric and cerebral arteries. Myogenic tone was impaired in middle‐aged mice and young mice with familial Alzheimer's mutations. Blocking the S1P_2_‐R/Rho‐kinase pathway had no age‐dependent effects. These findings offer new mechanistic insights into myogenic tone and its deficiencies in ageing and Alzheimer's disease.

## 1 INTRODUCTION

The normal function of critical organs relies on steady blood perfusion accomplished via autoregulatory mechanisms. Failure of blood flow autoregulation can lead to long‐term damage to cells and tissues, and eventually to organ failure with serious diseases or death as a consequence. One of the primary mechanisms of local blood flow autoregulation is the myogenic response in small arteries and arterioles (i.e., ‘resistance arteries’), in which an increased intraluminal pressure activates vasoconstriction, leading to increased local vascular resistance and a return of the blood flow to baseline levels observed before the pressure increase. Conversely, the myogenic response causes resistance arteries to dilate in the face of decreased intraluminal pressure, thereby returning flow to baseline levels. The effects of the myogenic response are (1) to secure nearly constant blood flow with supply of O_2_ and nutrients (and removal of CO_2_ and waste products), and (2) to protect the downstream capillaries from excessive flow and pressure causing them to rupture or develop peripheral oedema, or from insufficient flow causing interruption of capillary perfusion and ensuing tissue hypoxia. A third effect is setting the basal myogenic tone (MT) of resistance arteries, providing the set point from which neuro‐hormonal or local vasodilators/vasoconstrictors can adjust blood flow in organs to match their physiological demands (Davis et al., 2023; Hill et al., 2006; Jensen et al., 2017). Failure of blood flow autoregulation is associated with serious conditions such as stroke, heart failure, chronic kidney disease, neonatal disease states, and neurodegenerative diseases such as vascular dementia and Alzheimer's disease (Burke et al., 2014; Claassen & Zhang, 2011; Fang et al., 2024; Kroetsch & Bolz, 2013; Moshayedi & Liebeskind, 2021; Rhee et al., 2018; Xu et al., 2007). The therapeutic strategy for combating serious cardiovascular and cerebrovascular diseases may involve treatment with systemic vasodilators to reduce blood pressure or to reestablish organ blood flow in the face of excessive local vasoconstriction (Chen et al., 2016; Kokkoris et al., 2012; Konidala & Gutterman, 2004; Tomassoni et al., 2008). Whilst systemic vasodilators typically work through improvement of nitric oxide bioavailability, inhibiting Ca^2+^ channels, or blocking renin–angiotensin system effects (Godfraind, 2014; Mason & Cockcroft, 2006; Weir, 2007), they do not specifically restore the deficient autoregulation but rather compensate by reducing smooth muscle tone (Hill et al., 2009; Jensen et al., 2017). To develop new strategies to specifically intervene and correct deficiencies in the myogenic response, it is imperative to increase our knowledge about all signalling mechanisms involved in this response.

Despite intense research in the origins of the myogenic response since its discovery (Bayliss, 1902), we still do not have a complete and unequivocal elucidation of the local intrinsic mechanisms in the resistance artery wall causing myogenic response and maintenance of a constant basal (myogenic) tone. Whilst the endothelial cells may modify MT, a mechano‐sensitive mechanism within the smooth muscle layer in the vascular wall is postulated to sense the pressure and transduce a cellular signal into the vasomotor response. There are three main hypotheses to explain pressure sensing and transduction of the myogenic response (Davis et al., 2023): (1) activation of stretch‐sensitive ion channels causing pressure‐induced (myogenic) depolarisation and activation of voltage‐dependent Ca^2+^ channels, Ca^2+^ entry and smooth muscle contraction (Knot & Nelson, 1998; Kotecha & Hill, 2005; Welsh et al., 2002); (2) activation of mechano‐sensitive proteins in the extracellular matrix coupled to membrane‐spanning integrins to activate intracellular signal transduction pathways leading to depolarisation and Ca^2+^ entry or increased Ca^2+^ sensitivity of the contractile filaments (Ca^2+^ sensitisation) and increased actin polymerisation (Hill et al., 2009; Martinez‐Lemus et al., 2005; Sun et al., 2017, 2008; Walsh & Cole, 2013); and (3) mechano‐stimulation of G‐protein coupled receptors (GPCRs), either directly or via local release of receptor agonists, causing depolarisation and increased Ca^2+^ entry as well as Ca^2+^ sensitisation and actin polymerisation (Blodow et al., 2014; Brayden et al., 2013; Hoefer et al., 2010; Hui et al., 2015; Kauffenstein et al., 2016; Schleifenbaum et al., 2014; Schnitzler et al., 2008; Storch et al., 2015). The latter GPCR hypothesis quickly gained attention as it combines activation of the depolarisation/Ca^2+^ influx pathway with the Ca^2+^ sensitisation and actin polymerisation pathways (Davis et al., 2023). It is noteworthy that intrinsic, constitutive activation of some GPCRs enables them to become stimulated by mechanical cues, such as cell stretch or increased membrane tension. As such, the type 1 angiotensin receptor (AT_1_‐R) can be activated by cardiomyocyte stretch in a manner not dependent on local angiotensin II (Ang II) release or production (Zou et al., 2004). Mechano‐activation of AT_1_‐Rs may lead to canonical PLC_β_‐mediated and/or biased β‐arrestin‐mediated (e.g., via mitogen‐activated protein kinase (MAPK)/extracellular signal‐regulated kinase (ERK)) downstream signalling (Rakesh et al., 2010; Schnitzler et al., 2008; Zou et al., 2004). The inactive state of the AT_1_‐Rs can be stabilised by an inverse agonist, and this is the mechanism behind the actions of the ‘sartans’ (e.g., losartan, candesartan, valsartan) to inhibit constitutive activity of the AT_1_‐Rs (Qin et al., 2009; Yasuda et al., 2008; Zou et al., 2004). In humans, only one subtype of AT_1_‐R is expressed, whereas in rodents both an AT_1A_‐R and an AT_1B_‐R subtype are expressed. Interestingly, there are contradictory reports on the importance of the AT_1A_‐R subtype versus the AT_1B_‐R subtype in pressure sensation and the myogenic response (Blodow et al., 2014; Schleifenbaum et al., 2014). Only a few GPCRs were unequivocally shown to be intrinsically mechanosensitive (Davis et al., 2023; Zou et al., 2004), and the role of local release of vasoactive agonists to participate in pressure‐induced myogenic constriction has received limited attention (Davis et al., 2023). No single study has tested the relevance of all the proposed GPCRs in MT development using a universal protocol for evoking the myogenic response in mouse resistance arteries. However, this would be relevant due to the frequent application of murine knockout or transgenic models in cardiovascular research. Firstly, we aimed to perform a comprehensive study of the possible GPCRs involved in MT development in mouse small mesenteric arteries using pharmacological receptor antagonism as well as GPCR knockout mice. Secondly, we aimed to elucidate the possible age‐dependency of the pressure‐sensitive GPCR pathway involved in MT development since ageing induces profound vascular changes in molecular expression and regulatory mechanisms (Harraz & Jensen, 2021; Jensen, 2024), and is an important risk factor for the development of hypertension as well as all diseases associated with failed autoregulation of blood flow. A recent review found that ageing causes a reduction in myogenic tone with ageing (Jensen, 2024). Since reduced myogenic tone and brain blood flow autoregulation may cause vulnerability and ultimately disruption of the blood–brain barrier with ensuing neuroinflammation, ageing is now considered a risk factor for the development of Alzheimer's disease (Ungvari et al., 2021). Thus, in addition to the impact of chronological age, we furthermore aimed to investigate myogenic tone in a model of Alzheimer's disease. A recent proteomic study of the effects of ageing in small cerebral and mesenteric arteries from mice concluded that regulation of actin cytoskeleton and the RhoA/Rho kinase (ROCK) pathway was involved in the age‐dependent vascular dysfunction (Rabaglino et al., 2021). Since activation of GPCRs is coupled to activation of RhoA via G_α12_ activation, a further aim of our study was to investigate the link between a GPCR responsible for pressure sensation and initiation of MT with activation of the RhoA/ROCK pathway. Thus, the overarching objectives of the study were (1) to define a GPCR mainly responsible for pressure sensation in the development of MT, and (2) to elucidate if the identified GPCR pathway is subject to age‐dependent functional changes involving RhoA/ROCK activation that could be exploited for development of therapies targeted towards hypertension and/or vascular dysfunction in the elderly population.

## 2 METHODS

### 2.1 Ethical approval

All animal experiments were approved by the Danish Animal Experiments Inspectorate under the Danish Ministry of Justice (licence nos 2018‐15‐0201‐01465 and 2023‐15‐0201‐01379 to L.J.J., no. 2014‐15‐2935‐00048 to K.A.H., and no. 2020‐15‐0201‐00441 to B.I.A.), or the Animal Experiment Committee of the National Cerebral and Cardiovascular Center (approval no. 22042), and the JASRI Animal Use and Care Committee at SPring‐8 (approval nos. 2022B1230 and 2022A1310) in Osaka, Japan (to J.T.P.). All animal experiments were performed in compliance with the guidelines for the protection of animals used for scientific purposes (Directive 2010/63/EU). The authors confirm that this study complies with the animal ethical principles under which the journal operates.

### 2.2 Mice

Male C57BL/6J wild‐type mice in two age groups were used: young mice, 2–5 months old; middle‐aged mice, 11–14 months old. Wild‐type (WT) mice were purchased from Taconic Biosciences (Lille Skensved, Denmark) or Nihon SLC (Shizuoka, Japan). Young mice (BALB/c background) deficient in the purinergic receptor P2Y_2_ (6 males genotyped as P2Y_2_^−/−^) versus their wild‐type littermates (six males genotyped as P2Y_2_^+/+^) (Homolya et al., 1999) were bred in‐house from founder mice originally sourced from the University of North Carolina at Chapel Hill, Chapel Hill, NC (Dr R. Bouchet). Young mice (C57BL/6J background) deficient in the pyrimidinergic receptor P2Y_6_ (P2Y_6_^−/−^) versus their wild‐type littermates (P2Y_6_^+/+^) (two females/10 males genotyped as knock‐out (KO) or WT, respectively) (Bar et al., 2008) were bred in‐house from founder mice originally sourced from Université Libre de Bruxelles, Belgium (Dr B. Robaye). Finally, we used young (5 months old) transgenic 5xFAD familial Alzheimer's disease mice (5xFAD‐TG) versus their wild‐type littermates (5xFAD‐WT). Transgenic 5xFAD mice (TG (APPSwFlLon, PSEN1*M146L*L286V)6799Vas, Jax strain: 034840) were purchased from The Jackson Laboratory (Bar Harbor, ME, USA). These mice have a congenic background (C57BL/6J). Allele variants and background were confirmed by genotyping according to supplier protocols. Mice were housed in standard laboratory conditions with free access to normal chow and water, constant room temperature, humidity and a 12 h:12 h light–dark cycle. Mice were euthanised by cervical dislocation before harvesting tissues for pressure myography and qPCR. After the in vivo experiments, mice were euthanised by 100 mM KCl infusion to induce cardiac arrest.

### 2.3 Pressure myography

Pressure myography and MT measurements in mouse mesenteric arteries were performed according to published standard procedures (Björling et al., 2018, 2013; Jensen, 2025; Mikkelsen et al., 2016). In brief, third order mesenteric arteries were mounted between two glass pipettes in the pressure myograph chamber in which they were superfused with Krebs buffer (37°C; equilibrated with 95% O_2_–5% CO_2_; pH 7.40) and lumen perfused with Krebs buffer plus low‐endotoxin BSA (1%) at a pressure of 40 mmHg in the absence of lumen flow. A standard protocol involving acute constrictions to modified Krebs buffer with 75 mmol/L KCl (K75), noradrenaline (NA; 1 µM) and a 30 min pressure step (from 40 to 80 mmHg) was followed to ensure vessel vitality and reactivity. Preparations were excluded from further experimentation if they were either leaking (>1–2 mmHg pressure difference between the inlet and outlet pipettes), or did not constrict uniformly by >50% to K75 or >20% to NA, or if the resting (unstimulated) diameter was not stable over time due to vasomotion or vasospasm. The active pressure curve was constructed by incremental pressure steps from 20 to 120 mmHg, with 5 min between each step. Next, vessels were exposed to K75 once more, followed by adding the test drug of choice to the myograph chamber. All test drugs were applied at concentrations that did not inhibit the active constriction to K75 (as seen in preliminary experiments). After 10–20 min exposure to the test drug, another pressure curve was constructed (range 20–120 mmHg). Following washout of the test drug and resting for 10 min, vessel reactivity to K75 was tested again. Finally, vessels were superfused with Ca^2+^‐free Krebs buffer with EGTA for >20 min to ensure maximal dilation, and the final, passive pressure curve was constructed (range 20–120 mmHg).

### 2.4 qPCR

Quantitative real‐time PCR using total RNA extracted from isolated second and third order branches of mesenteric arteries and whole brain (or liver) from mice was performed as previously described in detail (Björling et al., 2018, 2013; Mikkelsen et al., 2016). In brief, primer pairs (forward and reverse) for each gene were designed through Primer Blast. The following primer set was used for mRNA quantification of *Agtr1a* (AT1_A_‐R): forward – GTTGCTGTGTCAGAGGGAGTTT; reverse – TGGCTTTCTTGGAGGGTTGC (218 bp; Acc. No. NM_177322.3). For *Agtr1b* (AT1_B_‐R) we used: forward – GGCAGCAGGGAGTAACAGA; reverse – TGGGGCAGTCATCTTGGATTC (214 bp; Acc. No. NM_175086.3). For *Cysltr1* (CYSLTR1) we used: forward – TGCTTCAGGGAGCAAAAGGA; reverse – ATCTGGTACCTCAGCACCTTTC (132 bp; Acc. No. NM_021476.5). For *P2ry2* (P2Y_2_‐R) we used: forward – CATTACGTGACTGTCCCGAGG; reverse – AGGACTCCGAGATCACGCTC (124 bp; Acc. No. NM_008773.4). For *P2ry4* (P2Y_4_‐R) we used: forward – ACCGGGTAGGAGTCTTGTCA; reverse – GCAGAACCTGGCTAGCTGAA (166 bp; Acc. No. NM_020621.4). For *P2ry6* (P2Y_6_‐R) we used: forward – GTTGCTGTGTCAGAGGGAGTTT; reverse – TGCCATTGTCCTGCTCCATAA (297 bp; Acc. No. NM_183168.2). For *S1pr2* (S1P_2_‐R) we used: forward – CAACTCCGGGACATAGACCG; reverse – CCAGCGTCTCCTTGGTGTAA (235 bp; Acc. No. NM_010333.4). For quantification of the reference gene *Actb* (β‐actin) we used: forward – AGCCATGTACGTAGCCATCC; reverse – CTCTCAGCTGTGGTGGTGAA (228 bp; Acc. No. NM_007393.3). The qPCR protocol (annealing at 60°C for 10 s; elongation at 72°C for 20 s) was performed using a LightCycler 480 apparatus (Roche Diagnostics A/S, Hvidovre, Denmark) with PCR Mastermix containing SYBR Green, primer pairs, and RT^+^ or RT^−^ samples from mesenteric arteries and brain (calibrator sample). Product size was the expected size for each gene, and no products were amplified in negative control samples (H_2_O; RT^−^). qPCR data were reported as $2^{-\Delta \Delta C_{T}}$, where ∆*C*_T_ = *C*_T_ (target) – *C*_T_ (*ActB*), and ∆∆*C*_T_ = ∆*C*_T_ (sample) – ∆*C*_T_ (calibrator) (Livak & Schmittgen, 2001).

### 2.5 Cell culture

Human coronary artery smooth muscle cells (hCASMC; no. C0175C; Thermo Fisher Scientific, Waltham, MA, USA) were cultured in Medium‐231 containing 5% of Smooth Muscle Growth Supplement (SMGS) (Thermo Fisher Scientific) and 50 U/mL penicillin, and 50 µg/mL streptomycin (no. A2212; Biochrom). Cells were maintained in an incubator at 37°C in a humidified atmosphere containing 5% CO_2_. Medium was changed every 48 h, and cells were used in passages 3–8 at 70–90% confluence. For experiments, cells were seeded at a density of 4000 to 8000 cells per cm^2^ and grown on sterile round glass coverslips (Ø 5 mm) placed at the bottom of six‐well plates.

### 2.6 Calcium imaging

Culture medium was removed by suction from wells containing hCASMCs growing on the round glass coverslips. Wells were rinsed twice with HEPES‐buffered physiological saline solution (HPSS) at room temperature, and a loading buffer with the ratiometric fluorescent Ca^2+^ indicator Fura‐PE3/AM was added to the wells. The loading buffer consisted of Fura‐PE3/AM (5 µM), 1% BSA (low‐endotoxin), 0.02% Pluronic F‐127, and 0.06% Cremophore EL dissolved in HPSS. Cells were exposed to loading buffer for 90–120 min at 32°C before the experiment started. After loading, coverslips with attached hCASMCs were transferred to the bottom of a heated perfusion chamber (Warner Instruments, Hamden, CT, USA, model PH‐1/RC‐22C) and superfused (1.5 mL/min) with HPSS (32°C; pH 7.40). Cells were superfused 20–30 min before an experiment to wash away excess dye outside the cells, and to allow intracellular esterase activity to trap Fura‐PE3 inside the cytoplasm by removing the AM‐groups. Ratiometric Ca^2+^ imaging of the Fura‐PE3 loaded cells was performed on an inverted fluorescence microscope (Olympus IX71, Olympus, Tokyo, Japan) equipped with a ×20 high‐quality quartz objective (Olympus UApo/340; NA = 0.75), a rapid filter switch light source (Oligochrome, T.I.L.L. Photonics, Graefelfing, Germany), filters for excitation at 340 nm vs. 380 nm and emission at ∼510 nm, and a high‐speed, high‐sensitivity cooled CCD camera (Sensicam QE, PCO, Kelheim, Germany). The data were acquired and subsequently analysed using Live Acquisition software (T.I.L.L. Photonics). Excitation was 100 ms at 340 nm, 70ms at 380 nm, and the sampling rate was 1 Hz. The output image size was 444 × 335 µm (resolution 688 × 520 pixels; 2 × 2 pixel binning), and 5–15 randomly chosen hCASMCs were analysed per experiment. Cells were pre‐incubated with JTE‐013 or vehicle (0.1 % dimethyl sulfoxide (DMSO)) for 10 min before starting an experiment. During Ca^2+^ imaging experiments, cells were superfused with the following HPSS buffers (containing either 5 µM JTE‐013 or vehicle): HPSS for 3 min to record the baseline; exposure to 50 µM UTP in normal HPSS (3 min); washout with Ca^2+^‐free HPSS (5 min); exposure to 50 µM UTP in Ca^2+^‐free HPSS (3 min); repeat exposure to 50 µM UTP in normal HPSS (3 min); washout with normal HPSS (2–3 min). This allowed offline determination of control UTP response (peak/plateau) in normal Ca^2+^‐containing solution; UTP response (peak/plateau) due to intracellular storage release of Ca^2+^ in Ca^2+^‐free solution, and rate of re‐entry of Ca^2+^ due to opening of storage‐activated (TRPC/Orai type) non‐selective Ca^2+^‐permeable channels (Alkhani et al., 2014; Inoue et al., 2006; Kim et al., 2013; Konig et al., 2013; Strobaek et al., 1996; Syyong et al., 2009; Wang et al., 2025). The rate of Ca^2+^ re‐entry was determined as the ∆*R*/∆*t* during the initial 30 s period after reintroducing extracellular Ca^2+^ in the presence of UTP and noting a visible increase in *R*.

### 2.7 Synchrotron radiation microangiography

We performed synchrotron radiation microangiography (SRM) measurements of cerebral arterial and arteriolar diameters in the anaesthetized mouse brain in vivo at BL20B2 of the synchrotron radiation facility SPring‐8 (Hyogo, Japan). Here, all young versus middle‐aged mice underwent surgical preparation during isoflurane inhalation anaesthesia (3–4% for induction and 1.5–2% for maintenance) for the SRM experiments as previously described (Katare et al., 2018; Shirai et al., 2013). The trachea was intubated for mechanical ventilation (PhysioSuite rodent ventilator, Kent Scientific (Torrington, CT), 6 µL/g tidal volume at ∼180 breaths/min) with oxygen‐enriched room air (40% O_2_). A jugular vein was cannulated with a polyurethane tube (1Fr) and flushed with heparinized saline (0.9% NaCl, heparin 50 IU/mL) and used subsequently for drug infusion. A tapered polyurethane cannula (C10PC‐MCA2A09, Instech Laboratories, Plymouth Meeting, PA) was then introduced into the right common carotid artery antegradely towards the brain for infusion of iodine contrast agent (100 µL bolus injection of Iomeron iodine 350 mg/mL, Bracco‐Eisai, Tokyo, Japan). Imaging was performed in a closed‐skull state on mice in a supine position so that the X‐ray beam (monochromatic radiation at 40 keV) passed through the circle of Willis and posterior cerebrum in a coronal plane. An X‐ray detector (C11440 ORCA Flash 4.0, Hamamatsu Photonics, Hamamatsu, Japan, with a 50 mm Nikkor lens) with a 200 µm GAGG scintillator was mounted 450 mm from the imaging sample stage. Micro‐angiogram sequences (cine‐scan) for ∼2 s (16‐bit format, 33 fps pixel size 4.52 µm) were recorded using HiPic 9.3 × 64 software (Hamamatsu Photonics). Baseline images collected in a cine‐scan were used to evaluate the diameter of cerebral vessels from the middle cerebral artery to penetrating arterioles. After baseline imaging, following intravenous infusion of vehicle, a bolus of the S1P_2_‐R inhibitor JTE‐013 (6 mg/kg body weight (BW)) was infused, and a repeat contrast agent infusion was imaged after 5 min of drug exposure. Following a washout period of 20 min, a bolus of the ROCK2 inhibitor KD025 (6 mg/kg BW) was infused, and after 5 min, a third scan was acquired. Finally, a bolus (4 mg/kg BW) of the calcium channel blocker nifedipine was infused to obtain diameter responses to a well‐known general vasodilator, and a last cine‐scan was acquired.

### 2.8 Solutions and chemicals

Krebs buffer for pressure myography was prepared with Milli‐Q water (resistivity 18.2 MΩ cm^2^ at 25°C) and consisted of the following (mmol/L): NaCl 118; KCl 4.7; MgSO_4_ 1.2; CaCl_2_·2H_2_O 2.0; NaHCO_3_ 25; KH_2_PO_4_ 1.2; d(+)‐glucose 5. Ca^2+^‐free Krebs buffer was the same as above, but with 2 mmol/L EGTA replacing CaCl_2_·2H_2_O. In High‐KCl (75 mmol/L; K75) Krebs, NaCl was replaced with equimolar amounts of KCl. All buffers for pressure myography were equilibrated with 95% O_2_–5% CO_2_ to adjust pH to 7.40 during experiments. HEPES‐buffered saline solution for Ca^2+^ imaging experiments contained (mmol/L): NaCl 140; KCl 5; MgCl_2_·6H_2_O 1.2; CaCl_2_·2H_2_O 2; HEPES 10; d(+)‐glucose 5. The content of nominally Ca^2+^‐free HPSS was similar, except that 2 mmol/L CaCl_2_ was replaced with 6 mmol/L mannitol to avoid changes in osmotic pressure. All drugs were acquired from Sigma‐Aldrich (Merck KGaA, Darmstadt, Germany) unless otherwise specified and prepared as stock solutions in Milli‐Q water or DMSO. Stock solutions were stored at −20°C and used within 2 months. The vehicle concentration never exceeded 0.1% in the recording chamber or myograph bath in any experiment. Infusion of drugs in vivo in SRM experiments was performed after dissolving drugs in a vehicle solution consisting of DMSO (1.33%), polyethylene glycol 300 (PEG‐300) (30%), and Tween‐80 (1%) in a sterile 0.9% NaCl solution (Han et al., 2020). DMSO and Tween‐80 were used at ∼30× lower concentrations than the threshold for behavioural effects in mice (Castro et al., 1995). We did not observe any adverse effects in mice after infusion of a bolus of vehicle in our SRM experiments.

### 2.9 Statistical analysis

Data were presented as mean ± SD (*N* = number of animals or cell culture batches/days; *n* = number of arteries or hCASMCs). Differences in a single parameter between two groups or treatments were evaluated using Student's *t*‐test, and differences between more than two groups or treatments were evaluated by two‐way ANOVA with or without Šidák's multiple comparisons test. *I* *n vivo* data for SRM were analysed using a mixed‐effects model with Dunnett's multiple comparisons test. GraphPad Prism software (v. 10; GraphPad Software, Boston, MA, USA) was used for graphical plotting and statistical testing. An α‐level of 0.05 was considered statistically significant.

## 3 RESULTS

**FIGURE 1:**
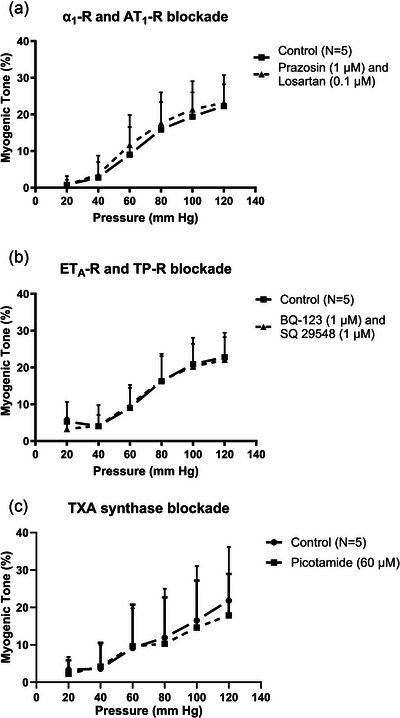
Myogenic tone in young mouse small mesenteric arteries before and during blockade of: α_1_‐R + AT_1_‐R using prazosin + losartan (a); ET_A_‐R + TP‐R using BQ‐123 + SQ 29548 (b); or thromboxane synthase using picotamide (c).

The initial set of MT experiments was performed to test whether local release of noradrenaline (NA), Ang II, endothelin‐1 (ET‐1), or thromboxane A2 (TXA_2_) is involved in MT development by using antagonists of their respective GPCR (α_1_‐R, AT_1_‐R, ET_A_‐R, TP‐R). In addition, we tested the effect on MT of the thromboxane synthase inhibitor picotamide. Since preliminary experiments did not show any effects of the receptor antagonists and to reduce the number of mice used, we exposed the vessels to a combination of prazosin and losartan, or a combination of BQ‐123 and SQ 29548, respectively. As shown in Figure 1a, b, neither of these exposures had any effects on MT development in mouse small mesenteric arteries. We did not test high concentrations of losartan to exploit inverse agonist properties of this drug, since preliminary experiments showed that higher losartan concentration blocked the KCl‐induced vasoconstrictions. Picotamide exposure did not have any effect, suggesting that local release of thromboxanes is not involved in MT (Figure 1c).

**FIGURE 2:**
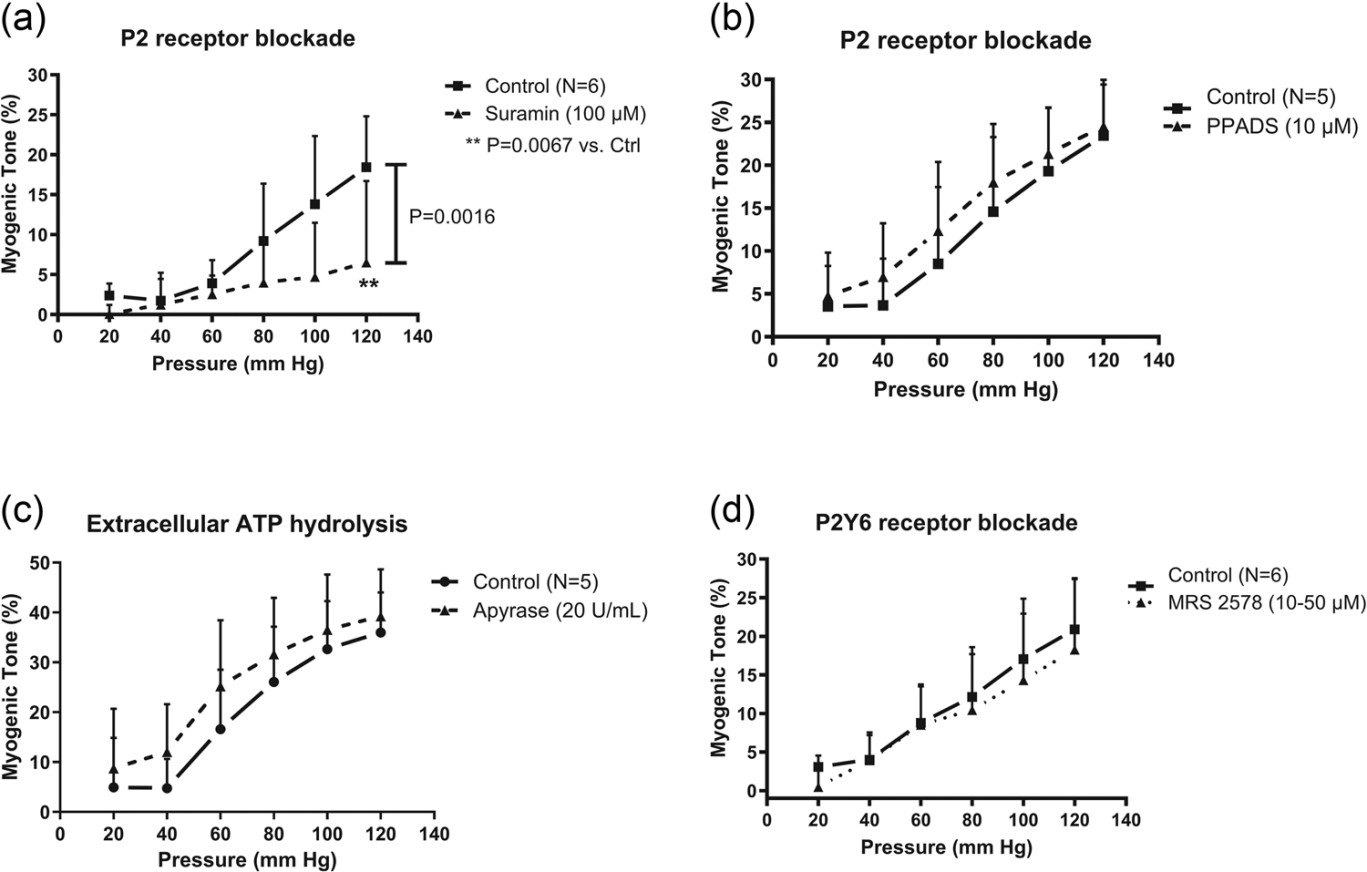
Myogenic tone in young mouse small mesenteric arteries before and during blockade of: P2‐receptors using suramin (a) or PPADS (b); quenching of extracellular ATP using apyrase from potato (c); or inhibition of P2Y_6_‐receptors using MRS 2578 (d). Statistically significant difference between two curves (main effect) shown as *P*‐values next to vertical bar (in (a)). Significant differences at each pressure level in control versus suramin are shown with asterisks.

Our next aim was to test the involvement of local release of purines or pyrimidines as a mechanism to induce MT development via activation of purinergic or pyrimidinergic GPCRs. For this purpose, we exposed the vessels to two chemically unrelated non‐specific purinergic (P2‐) receptor blockers: suramin or 4‐[[4‐formyl‐5‐hydroxy‐6‐methyl‐3‐[(phosphonooxy)methyl]‐2‐pyridinyl]azo]‐1,3‐benzenedisulfonic acid tetrasodium salt (PPADS). The experiments showed differential effects, namely a robust inhibition of MT by suramin, but in contrast, no effects of PPADS (Figure 2a, b). Since suramin is known to inhibit vasointestinal peptide (VIP) receptors by physically disrupting receptor–G protein coupling (Chung & Kermode, 2005), we also tested the effects of quenching extracellular ATP released from the vessels using the enzyme apyrase. However, no effects were seen on MT by apyrase exposure (Figure 2c). Since pyrimidinergic P2Y_6_‐R are known to elicit strong vasoconstrictions to UDP in human cerebral vasculature (Malmsjo et al., 2003), we also tested the small‐molecule P2Y_6_‐R inhibitor MRS 2578 on the MT. We tested two high concentrations of the drug (10 and 50 µmol/L) with no effects, and we have thus pooled the data in Figure 2d.

**FIGURE 3:**
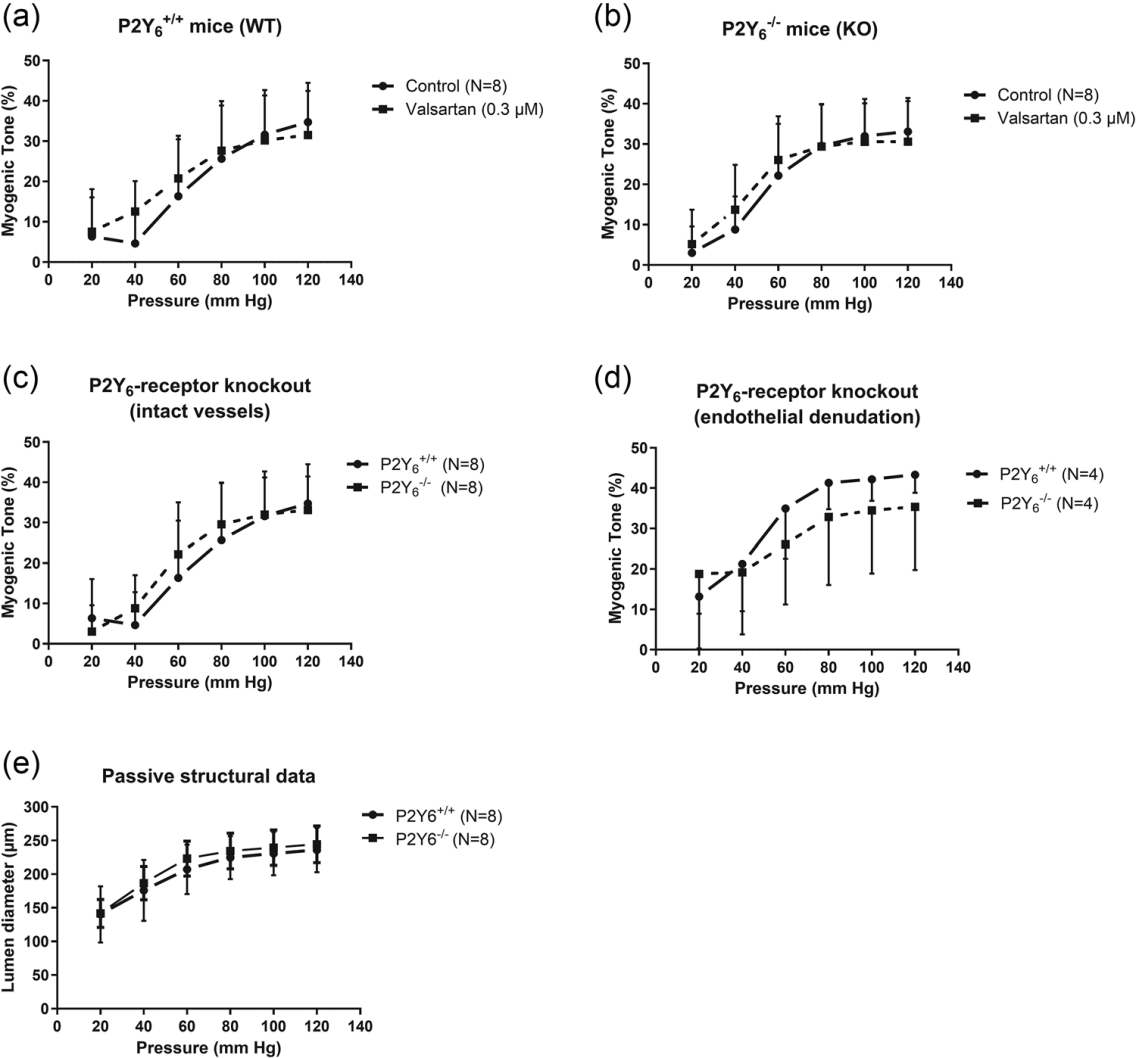
Myogenic tone and passive lumen diameters in mouse small mesenteric arteries in young mice deficient in P2Y_6_‐Rs. (a, b) Effect of AT_1_‐R blocker valsartan in WT (a) versus KO (b) mice. (c) Comparison of control MT in age‐matched WT versus KO mice. (d) Effect of endothelial denudation in age‐matched WT versus KO mice. (e) comparison of passive structural diameter in age‐matched WT versus KO mice.

**FIGURE 4:**
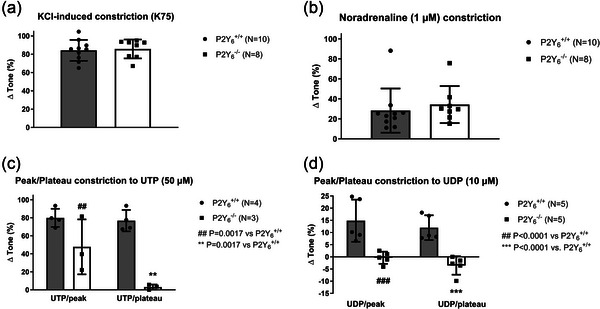
Vasoconstriction (%, ∆Tone) to K75 (a), NA (b), UTP (c), and UDP (d) in isolated mesenteric arteries from age‐matched P2Y_6_‐R WT versus KO mice.

However, as P2Y_4_‐R and P2Y_6_‐R were implicated in MT in cerebral vessels (Brayden et al., 2013), we decided to test MT in small mesenteric arteries from mice with a global knockout of P2Y_6_‐R. In the first set of experiments with these mice, we also decided to test the effects of valsartan, which is a newer generation of AT_1_‐R antagonist, as this might reveal an involvement of AT_1_‐Rs in MT development. However, there were no effects of valsartan in wild‐type (P2Y_6_^+/+^) or knockout (P2Y_6_^−/−^) mice (Figure 3a, b). Furthermore, there was no difference between MT (before valsartan) between P2Y_6_^+/+^ versus P2Y_6_^−/−^ (Figure 3c). The possibility remained that the endothelium could release a paracrine factor that masks the effect of the P2Y_6_‐R knockout in the smooth muscle cells, so we repeated the comparisons in the P2Y_6_‐R mice this time using endothelium‐denuded small mesenteric arteries. Nevertheless, endothelium denudation did not unmask any significant differences in MT between the P2Y_6_^+/+^ and P2Y_6_^−/−^ mice (Figure 3d). The passive lumen diameters were not different in the P2Y_6_‐R KO versus WT mice, showing that P2Y_6_‐Rs are not crucially involved in structural vascular remodelling (Figure 3e). Finally, we tested the vasoconstrictor properties of exposure to high‐KCl, NA, UTP and UDP to ensure that our knockout mice had the relevant phenotype. As expected, we did not see any difference in vasoconstriction to high‐KCl or NA (Figure 4a, b). The peak response to UTP was partially reduced, and the plateau UTP response was abolished in the P2Y_6_^−/−^ mice (Figure 4c). During exposure to UDP, both peak and plateau vasoconstrictions were abolished in the P2Y_6_^−/−^ mice, as expected (Figure 4d). The remaining peak of the UTP response in the P2Y_6_^−/−^ mice might be induced by transient activation of purinergic receptors, such as P2Y_2_‐R (Haanes et al., 2016). Taken together, these data indicate that P2Y_6_‐Rs were effectively absent in small mesenteric arteries in P2Y_6_^−/−^ mice, and yet we observed no change in MT.

**FIGURE 5:**
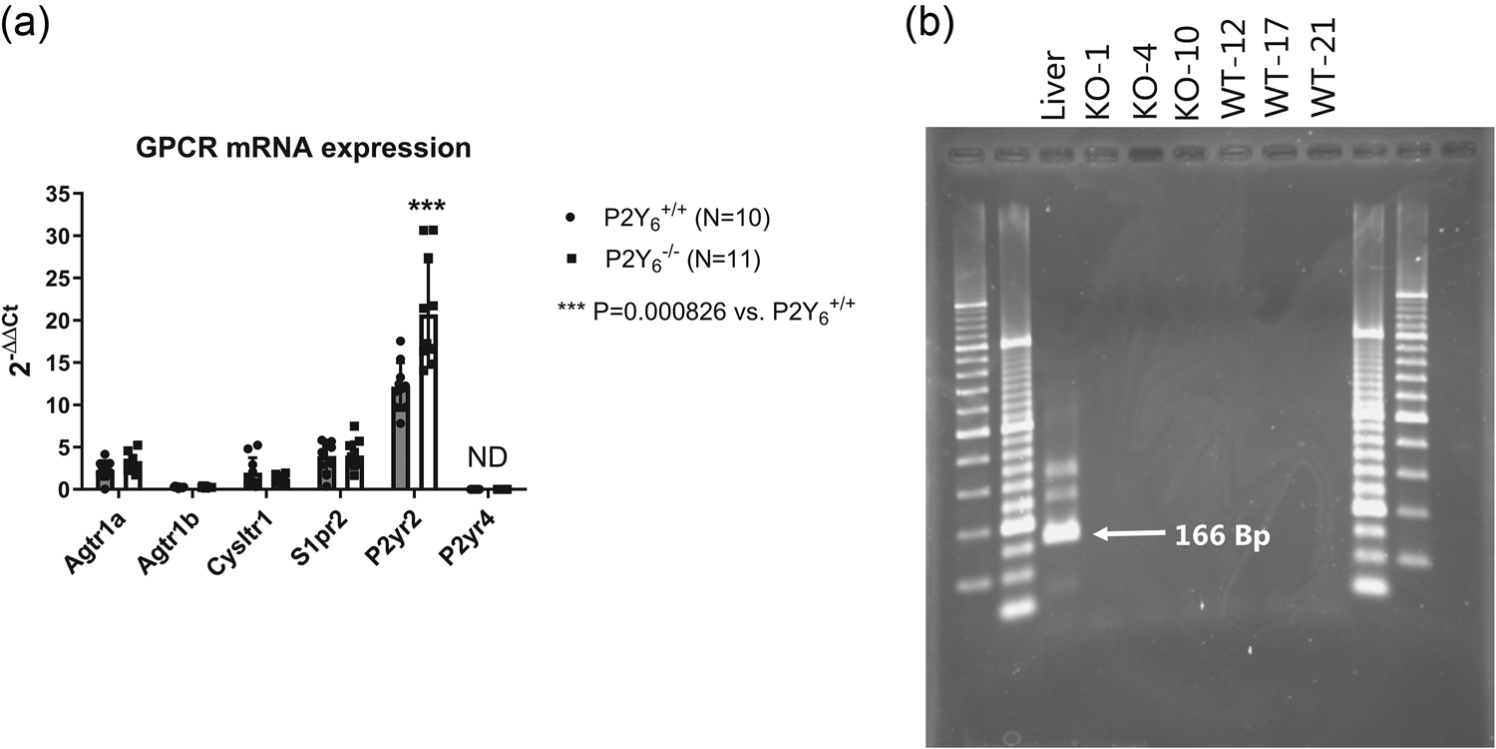
(a) Comparison of mRNA expression of various GPCRs putatively involved in pressure‐sensing and MT development in small mesenteric arteries in age‐matched P2Y_6_‐R WT versus KO mice. (b) DNA electrophoresis gel showing the absence of bands corresponding to the product of P2Y_4_‐Rs in three P2Y_6_‐R KO and three WT mice, using a liver sample as a positive control. Two different molecular size ladders are shown to the left and right.

**FIGURE 6:**
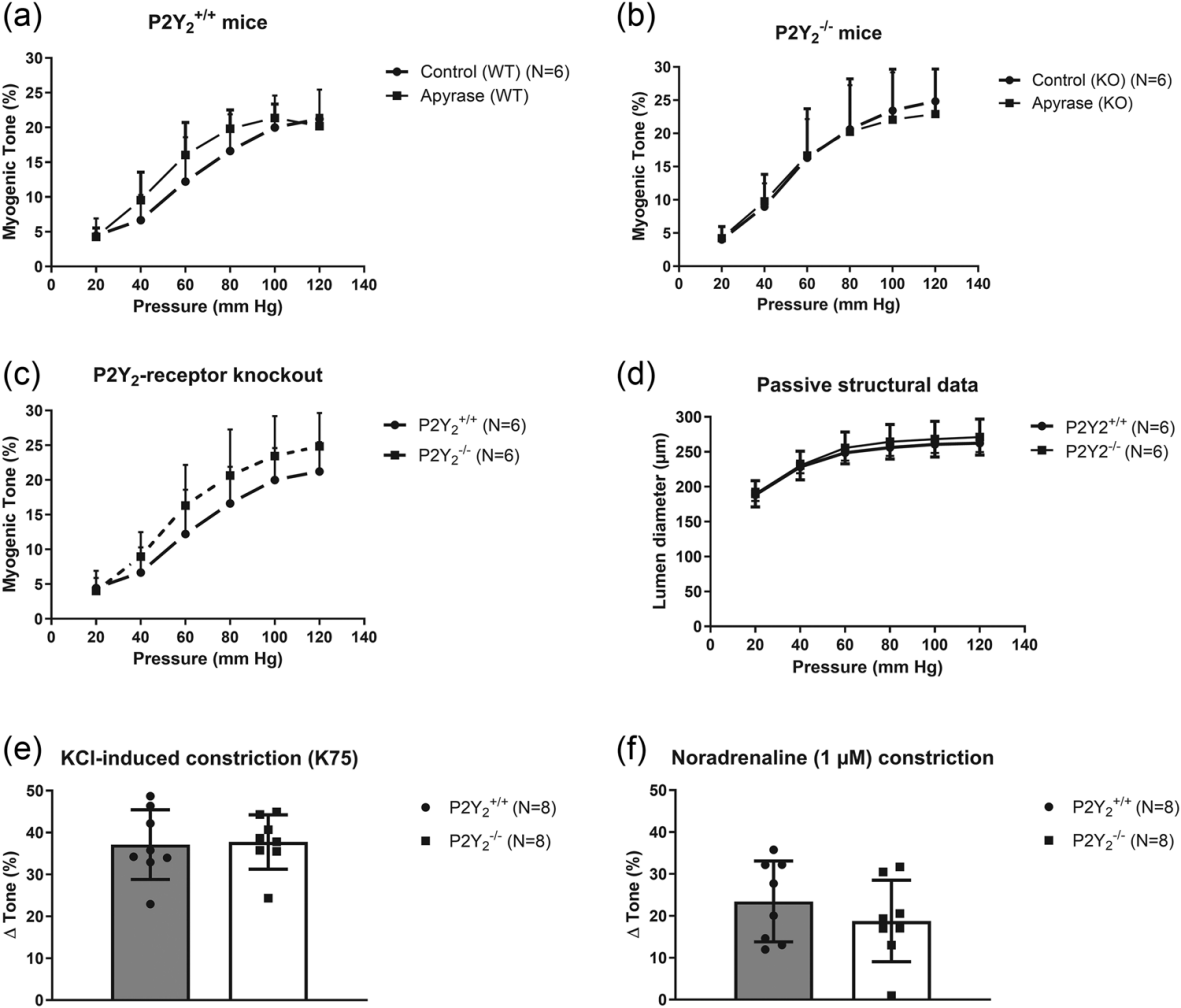
Myogenic tone, vasoconstriction and passive lumen diameters in mouse small mesenteric arteries in young mice deficient in P2Y_2_‐Rs. (a, b) Effect of apyrase in age‐matched WT (a) versus KO (b) mice. (c) Comparison of control MT in age‐matched WT versus KO. (d) Comparison of passive lumen diameters in age‐matched WT versus KO. (e, f) Vasoconstrictions to K75 (e) and NA (f) in age‐matched WT versus KO mice.

We also tested whether knockout of P2Y_6_‐R caused compensatory gene expression changes in the GPCRs previously suggested to be involved in MT. Figure 5a shows that we did not detect any differences between P2Y_6_‐R WT and KO animals in small mesenteric artery mRNA levels of AT_1_‐R transcripts (*Agtr1a*; *Agtr1b*), cysteinyl leukotriene receptor 1 (*Cysltr1*), or sphingosine 1‐phosphate receptor 2 (*S1pr2*). However, we did find a robust and highly significant upregulation of P2Y_2_‐receptor (*P2ry2*). Interestingly, we did not detect any expression of P2Y_4_‐R (*P2ry4*) (Figure 5a) as confirmed by the lack of *P2ry4* amplification in the DNA gel electrophoresis blot (Figure 5b). This absence of P2Y_4_‐R expression is in line with previous findings from human small omental and cerebral arteries (Malmsjo et al., 2003), but in contrast to findings from rat cerebral parenchymal arterioles (Brayden et al., 2013). The upregulation of *P2ry2* prompted us to investigate the potential role in MT using global P2Y_2_‐R knockout mice, and in addition, verify the lack of an effect of ATP quenching using apyrase. Our data confirmed the absence of an apyrase‐induced effect on MT in both WT and KO animals (Figure 6a, b). Importantly, there were no differences in MT (before apyrase) between the P2Y_2_^+/+^ and P2Y_2_^−/−^ mice (Figure 6c), demonstrating that, despite an upregulation of P2Y_2_‐R in P2Y_6_‐R‐deficient mice, MT development in small mesenteric arteries is not functionally dependent on expression of P2Y_2_‐R. The passive lumen diameters in the wild‐type and knockout P2Y_2_‐R mice were not different, showing that P2Y_2_‐Rs are not likely to participate in structural vascular remodelling (Figure 6d). As expected, we did not see any difference in vasoconstriction to high‐KCl or NA in the P2Y_2_‐R mice (Figure 6e, f). Overall, P2Y_6_‐R, P2Y_4_‐R and P2Y_2_‐R are therefore not involved in MT development in mouse small mesenteric arteries.

**FIGURE 7:**
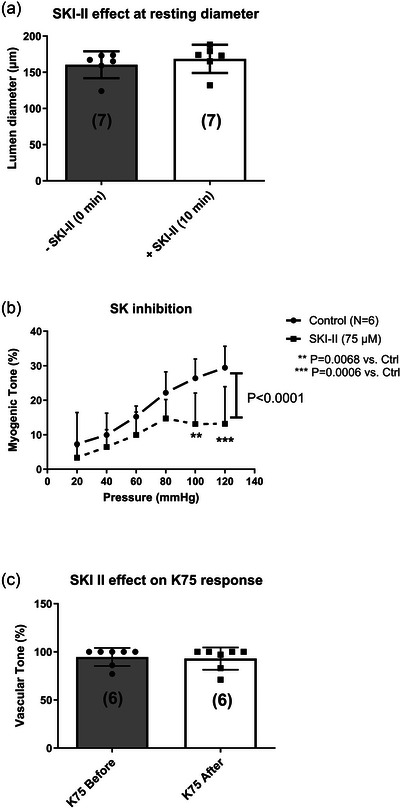
Resting diameter at 40 mmHg (a), myogenic tone (b), and vasoconstriction to high‐KCl (c) in young mouse small mesenteric arteries before and after addition of 75 µmol/L SKI II to the pressure myograph bath. Number of mice shown in brackets (a, c). Statistically significant difference between two curves (main effect) shown as *P*‐values next to vertical bar (in (b)). Significant differences at each pressure level in control versus SKI II are shown with asterisks.

Prompted by a rather high mRNA expression of S1P_2_‐R (S1pr2) in mesenteric arteries (Figure 5a), we decided to pharmacologically target S1P_2_‐Rs and sphingosine‐1‐phosphate (S1P) production via sphingosine kinase (SK). Using the selective SK1/2 inhibitor SKI‐II, we did not observe an effect on the resting diameter of small mesenteric arteries pressurised at 40 mmHg (Figure 7a), but MT development at higher pressures was significantly blunted by the inhibitor (Figure 7b). The effect of SK inhibition was specific for MT, as we did not find an inhibitory effect of SKI‐II on KCl‐induced vasoconstriction (Figure 7c).

**FIGURE 8:**
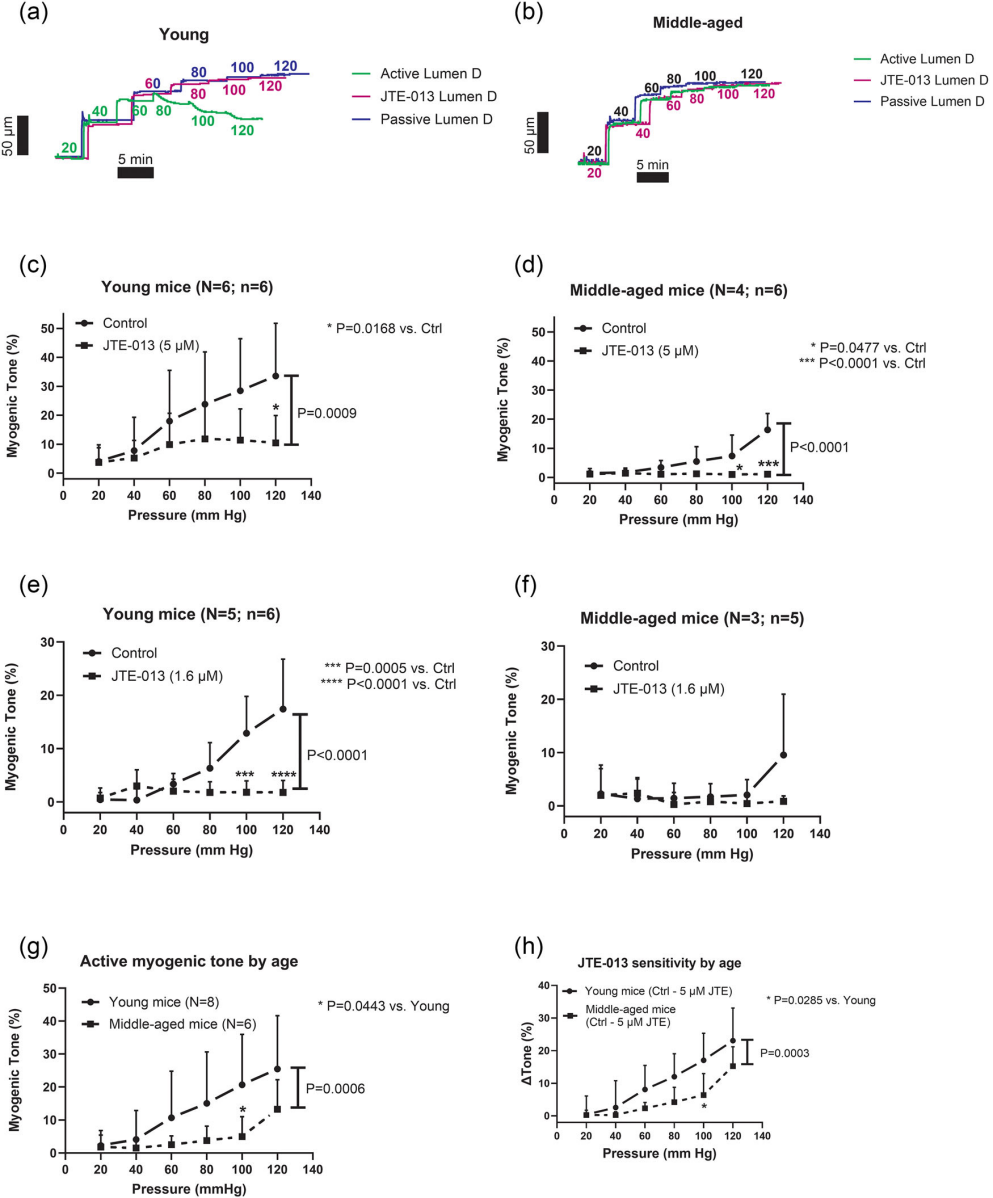
(a, b) Raw lumen diameter tracings of myogenic responses (green, active) recorded in small mesenteric arteries from young (a) versus middle‐aged (b) mice. Pressure curve with S1P_2_‐receptor blocker JTE‐013 (purple) and passive pressure curve (blue) are also shown in (a, b). Numbers adjacent to the curves indicate the intraluminal pressure. (c–f) Myogenic tone in young and middle‐aged mouse small mesenteric arteries before and during blockade of S1P_2_‐receptors using JTE‐013 at two concentrations (1.6 and 5 µmol/L). (g) Comparison of control (active) MT in young versus middle‐aged mice. (h) Comparison of the sensitivity (control MT *minus* JTE MT) to 5 µmol/L JTE‐013 in young versus middle‐aged mice. Statistically significant difference between two curves (main effect) is shown as *P*‐values next to the vertical bar. Significant differences at each pressure level in control versus JTE‐013 are shown with asterisks.

**FIGURE 9:**
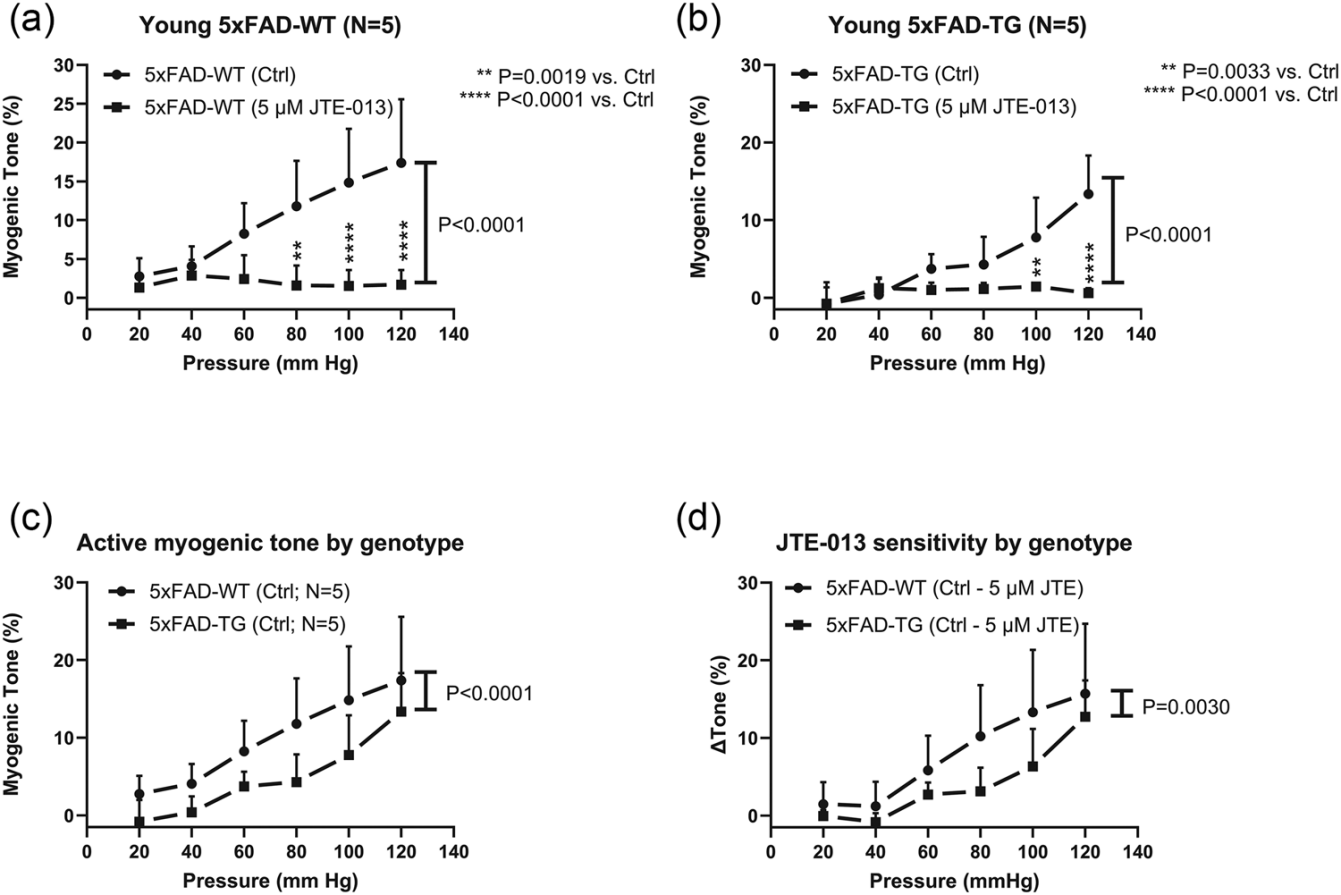
(a, b) Myogenic tone in small mesenteric arteries from young 5xFAD WT (a) versus TG (b) before and during blockade of S1P_2_‐receptors using 5 µmol/L JTE‐013. (c) Comparison of control (active) MT in 5xFAD‐WT versus ‐TG mice. (d) Comparison of the sensitivity to 5 µmol/L JTE‐013 in 5xFAD‐WT versus ‐TG mice. Statistically significant difference between two curves (main effect) is shown as *P*‐values next to the vertical bar. Significant differences at each pressure level in control versus JTE‐013 are shown with asterisks.

We next aimed to test if S1P_2_‐R is involved in MT by exposing the vessels to the only available small molecule S1P_2_‐R inhibitor, JTE‐013. In addition, we wanted to investigate if S1P_2_‐R inhibition has age‐dependent effects by testing MT in young versus middle‐aged mice. Using JTE‐013 at a concentration (5 µmol/L) that did not acutely inhibit KCl‐induced vasoconstriction in preliminary experiments, we observed that JTE‐013 nearly abolished MT in both young and middle‐aged mouse mesenteric arteries (Figure 8a–d). At this point, we observed that myogenic reactivity was much reduced in the middle‐aged animals (Figure 8b, d). To test if we could detect an age‐dependent drug effect at lower concentrations, we performed a new set of experiments with 0.5Log[JTE] lower concentration, that is, at 1.6 µmol/L (Figure 8e, f). However, even at this lower concentration, we observed near abolishment of MT in mesenteric arteries from both age groups. For practical reasons, we could not perform another set of experiments with even lower JTE‐013 concentrations. Our data show that MT was significantly impaired in the middle‐aged mice (Figure 8g), and that there was a significant difference between the JTE‐013‐induced MT inhibition (Δ Tone%) between young and middle‐aged mice (Figure 8h). However, we infer that the weaker effect of JTE‐013 in middle‐aged mice can be explained by the reduced MT at this age. Next, we explored the possible role of MT and S1P_2_‐R inhibition in another age‐related mouse model, the 5xFAD model of familial Alzheimer's disease. The data showed that JTE‐013 (5 µmol/L) nearly abolished MT in 5‐month‐old male 5xFAD‐transgenic mice (5xFAD‐TG) and in their littermate control animals (5xFAD‐WT) (Figure 9a, b). The MT was significantly lower in 5xFAD‐TG compared to their wild‐type littermates (Figure 9c), suggesting that Aβ deposition, oxidative stress and inflammatory signals associated with AD pathology in these mice can somehow impair the function of systemic resistance arteries. Finally, the effect of JTE‐013 on MT (Δ Tone%) was significantly weaker in the 5xFAD‐TG mice (Figure 9d), but this is most likely due to a lower baseline MT in the transgenic AD mice.

**FIGURE 10:**
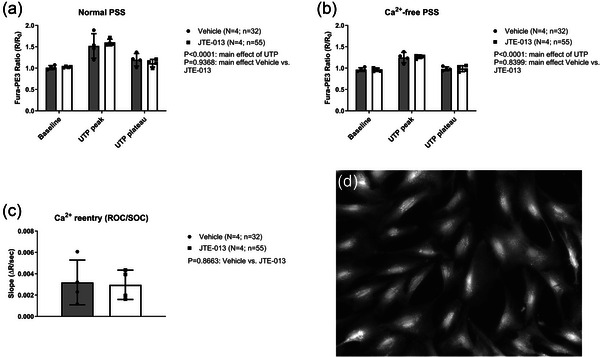
(a, b) Intracellular Ca^2+^ responses (baseline, peak and plateau) in hCASMCs to exposure with UTP (50 µmol/L) in normal HPSS with Ca^2+^ (a) and in nominally Ca^2+^‐free HPSS with 5 µmol/L JTE‐013 or vehicle (DMSO, 0.1 %) (b). (c) The rate of change in Fura‐ratio after bath re‐entry of extracellular Ca^2+^ in the continued presence of UTP (with JTE‐013 or vehicle), which is an indicator of Ca^2+^ entry via receptor/storage‐activated non‐selective cation channels (ROC/SOC; TRPC/Orai channels). In (a–c), *N* denotes the number of cultured hCASMC batches; *n* denotes the number of individual hCASMCs. (d) Image of Fura‐loaded hCASMCs showing Fura‐emission at ∼510 nm during excitation with 380 nm UV light. Image size 444 × 335 µm.

Since previous research suggested that JTE‐013 may have off‐target effects on other S1P‐receptors or on sphingosine metabolism (Pitman et al., 2022; Salomone et al., 2008), we questioned whether JTE‐013 could inhibit MT indirectly by affecting dynamic intracellular calcium responses in vascular smooth muscle cells. We tested this by comparing the intracellular Ca^2+^ responses to UTP exposure in hCASMCs preincubated with either 5 µmol/L JTE‐013 or vehicle. As shown in Figure 10a–c, JTE‐013 preincubation did not affect the peak or plateau Ca^2+^ responses to UTP in the presence or absence of extracellular calcium, nor did it affect the rate of re‐entry of Ca^2+^ into hCASMCs via receptor‐/storage‐operated Ca^2+^ channels. In preliminary experiments, we did not detect any effects on MT by exposing small mesenteric arteries to the S1P_3_‐R blocker TY‐52156. These results indicate that the effects of JTE‐013 on MT are not due to the unspecific impacts on Ca^2+^ signalling pathways in the mouse small mesenteric arteries, and most likely are caused by inhibition of S1P_2_‐Rs.

**FIGURE 11:**
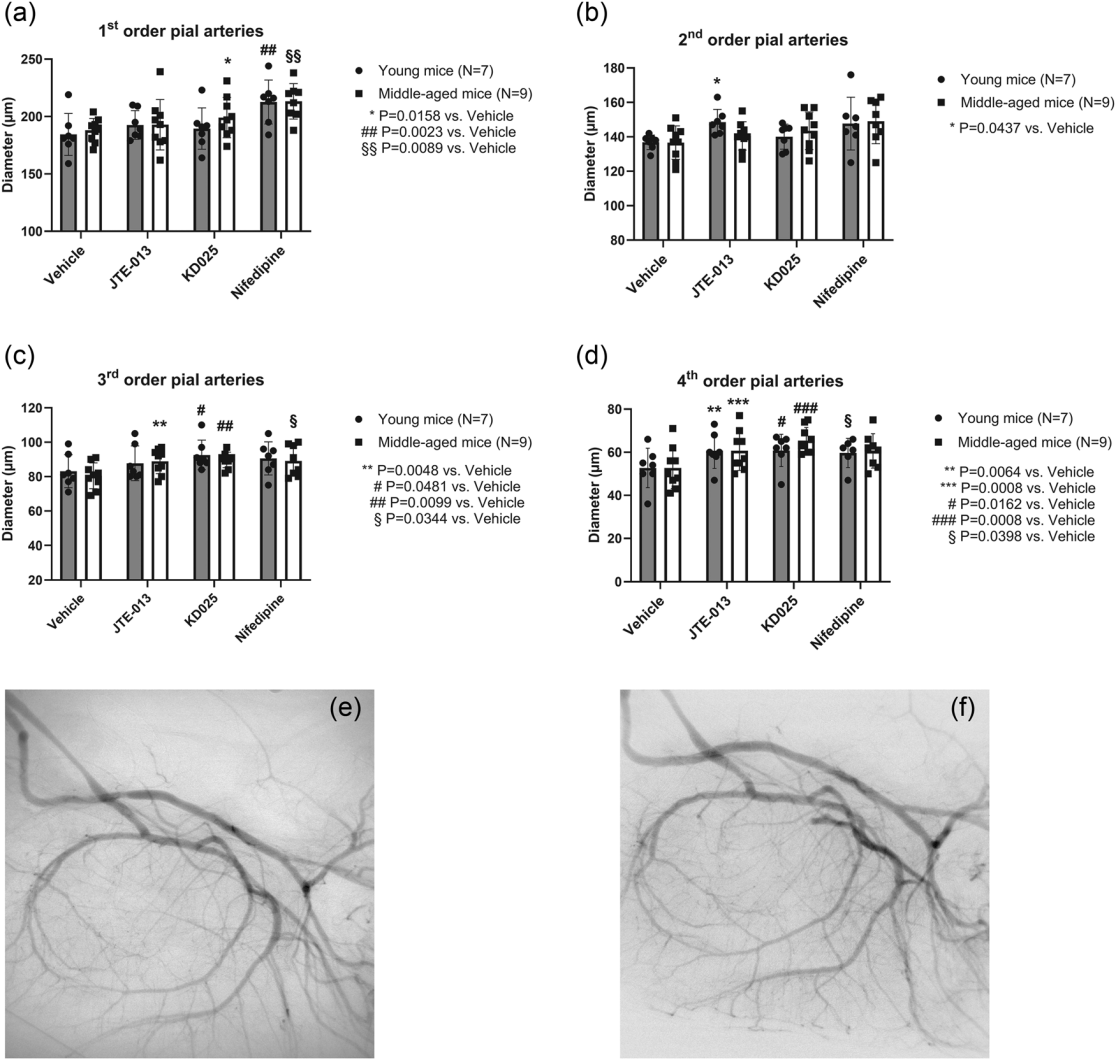
Synchrotron radiation microangiography measurements of the resting diameters of the arterial circulation (1^st^–4^th^) branches of cerebral arteries from the main branch (zero order). (a–d) Diameters during exposure to vehicle, a bolus with the S1P_2_‐R blocker JTE‐013, a bolus with the ROCK2 blocker KD025, or a bolus with the L‐type channel blocker nifedipine. (e, f) SRM images (9257 × 9257 µm) of the cerebral arterial circulation obtained during iodine‐based contrast medium infusion in the brains of a young (e) and a middle‐aged (f) mouse (both exposed to vehicle).

**FIGURE 12:**
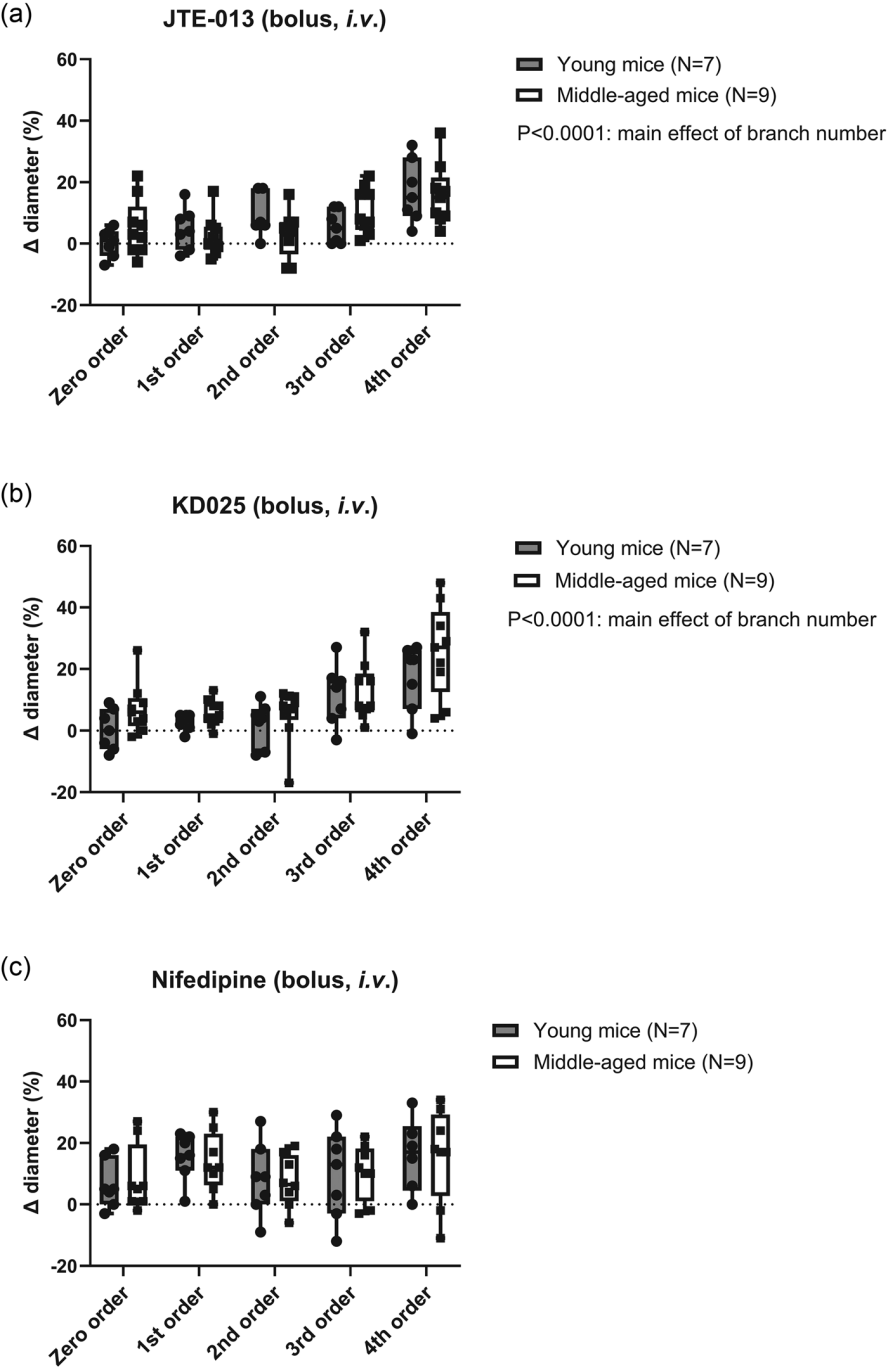
Changes (%, means ± SD) relative to vehicle in cerebral arterial 1^st^–4^th^ order branch diameter (∆ diameter) to a bolus exposure with either JTE‐013 (a), KD015 (b) or nifedipine (c) in young versus middle‐aged mice. The main effect of branching order indicates a statistically significant difference between small versus large diameter branches.

**FIGURE 13:**
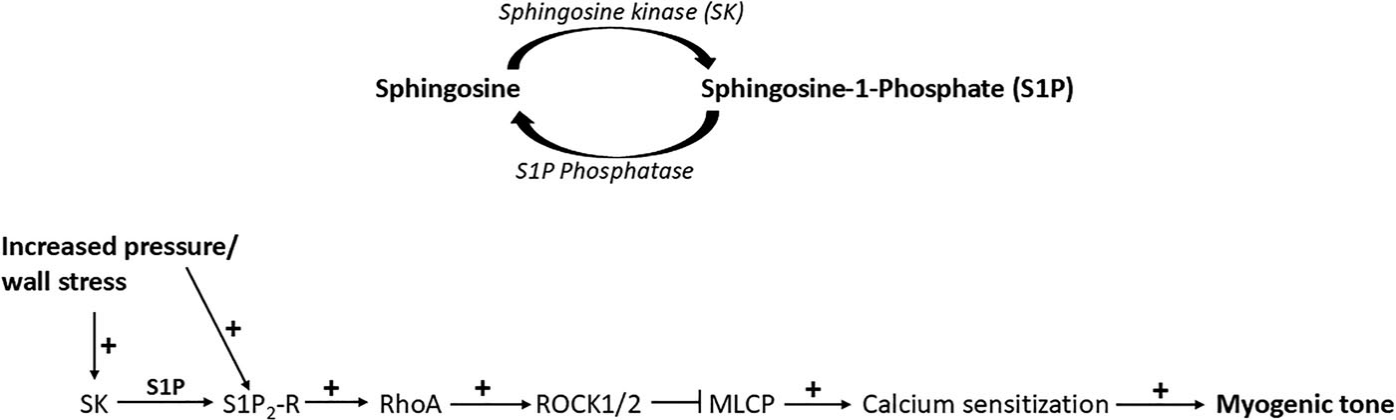
Suggested model for how S1P signalling interacts with and sustains myogenic tone development in the wall of resistance arteries, via activation of the RhoA/ROCK pathway and calcium sensitisation. At present, it is not known how increased wall stress activates sphingosine kinase. A part of the myogenic tone developed was not sensitive to SK inhibition, but still sensitive to S1P_2_‐R receptor inhibition, suggesting that S1P_2_‐R can also be directly activated by increased wall stress. When myogenic tone is increased (i.e., radius decreased), the wall stress is reduced (assuming no change in arterial pressure), thus providing a negative feedback control on the activation of this pathway.

To test the functional role of S1P_2_‐Rs in vivo and to assess the possible role of age‐dependent effects, we performed synchrotron radiation microangiography on the mouse brain arterial circulation in young versus middle‐aged mice. In addition to testing intravenous bolus infusions of JTE‐013 on resting cerebrovascular diameter, we also aimed to clarify the role of Rho‐kinase 2 (ROCK2) using the specific ROCK2 inhibitor KD025 (Björling et al., 2018), and to compare these effects with inhibition of the arterial L‐type Ca^2+^ channels using nifedipine. The data were evaluated for first to fourth order branches of the middle cerebral arteries, thus allowing for discrimination between effects on proximal cerebral arteries versus more distal (penetrating) arterioles. The overall data showed that nifedipine largely dilates all branches from first to fourth order (Figure 11a–d), whereas JTE‐013 and KD025 only dilate the more distal third and fourth order arteries (Figure 11c, d). Since the more distal systemic arteries are known to possess higher myogenic reactivity than more proximal branches, this observation is consistent with our hypothesis that S1P_2_‐R and ROCK are involved in setting the basal tone. Moreover, there were no age‐dependent differences in the resting diameters during vehicle or drug infusions. When the individual drug effects were evaluated as percentage change in diameter relative to vehicle (Figure 12a–c), statistical comparisons likewise indicated that the JTE‐013 and KD025 effects were dependent on branching order (i.e., effects are significantly different in small vs. large branches), and there were no age‐dependent differences. A model with the proposed role of SK and S1P_2_‐R in myogenic tone development in small arteries is shown in Figure 13.

## 4 DISCUSSION

The pressure sensing involved in the myogenic activity of resistance arteries is still not well understood despite intensive research. One problem with defining the initial molecular pressure‐sensing step is the inherent difficulty of excluding parallel pressure‐sensing steps (i.e., redundancy) or other upstream pressure sensors triggering the mechanism under study. Therefore, each new candidate pressure sensor mechanism in the myogenic response must be challenged by other investigators using different methods and vascular beds to allow for a complete characterisation of its universal role. The present study was undertaken to critically evaluate current hypotheses regarding the involvement of GPCR activation as an initial or upstream event in the myogenic response signalling. Previous studies focused on a diversity of mechanisms and vascular beds, but here we have sought to eliminate unnecessary bias and variation due to different experimental approaches and vascular beds by using a universal experimental protocol applied in third order mouse mesenteric arteries.

As no effects on MT were seen by blocking α_1_‐, AT_1_‐, ET_A_‐, and TP‐receptors with therapeutically relevant antagonist concentrations, our study provided unequivocal evidence that local release of agonists to these GPCRs is not involved in initiating or modifying MT. Activation of purinergic (P2X or P2Y) receptors by local ATP release can also be excluded, as we found no effect of quenching extracellular ATP using apyrase. In the case of AT_1_‐Rs, it may be difficult to exclude the possibility that they have constitutive (low) G_α_‐protein or β‐arrestin activity, which can be augmented by a mechanical (pressure) stimulus. We used the antagonists losartan and valsartan at therapeutically relevant concentrations (0.1 to 0.3 µmol/L), but since both drugs tend to inhibit KCl‐induced vasoconstriction when used at higher concentrations in the micromolar range, we could not test the inverse agonist properties at higher drug concentrations. Furthermore, there are conflicting reports as to which of the two AT_1A_‐R or AT_1B_‐R subtypes might be involved in MT development in mice (Blodow et al., 2014; Schleifenbaum et al., 2014). Taken together, these observations suggest that more experiments with higher translational value are needed to ascertain the role of AT_1_‐Rs in MT development.

We can make a firm conclusion that purinergic or pyrimidinergic P2Y_2_‐, P2Y_4_‐ and P2Y_6_‐Rs are not involved in MT development in mouse mesenteric small arteries: (1) because P2Y_4_‐Rs were not expressed in the arteries, (2) because global knockout of P2Y_2_‐R did not affect MT, and (3) a thorough characterisation of vascular responses in mice deficient in P2Y_6_‐Rs showed no differences in MT compared to WT in endothelium‐denuded and ‐intact vessels. In addition, no differences in NA‐induced constriction were observed in mice deficient in either P2Y_2_‐R or P2Y_6_‐R. The latter result deserves attention due to previous studies showing that pannexin 1‐mediated ATP release augments α_1_‐R‐mediated noradrenaline constriction (Billaud et al., 2011), and since P2Y_2_‐Rs and P2Y_6_‐Rs can heterodimerise or functionally interact with adenosine A_1_‐ and angiotensin AT_1_‐rceceptors (Daghbouche‐Rubio et al., 2024; Namba et al., 2010; Nishimura et al., 2016). If such interaction mechanisms enabled P2Y_2_‐ or P2Y_6_‐Rs to establish crosstalk with α_1_‐Rs, we would expect that an absolute deficiency of P2Y_2_‐ or P2Y_6_‐R expression would affect NA‐induced vasoconstriction negatively. As we did not observe such an effect, it means that we can rule out a major role for interaction and crosstalk between the pyrimidinergic receptors and α_1_‐Rs.

In mice deficient in the ectonucleotidase NTPDase1, UTP‐ and UDP‐induced vasoconstriction was robustly augmented in aortic rings, and MT was enhanced in small mesenteric arteries as compared to what seems an unusually low MT measured in WT arteries (Kauffenstein et al., 2010). In a later study, this group published that UTP‐ and UDP‐induced contractions in small mesenteric arteries were abolished in mice deficient in P2Y_6_‐Rs (Kauffenstein et al., 2016). Furthermore, MT was nearly abolished in small mesenteric arteries from P2Y_6_‐R KO mice. However, the control MT curve in WT littermates was bell‐shaped as the MT peaked at 50 mmHg and declined sharply at higher pressures (Kauffenstein et al., 2016), unlike MT that normally increases with pressure in mouse mesenteric arteries (Björling et al., 2018, 2013; Mikkelsen et al., 2016) (and present study). Our results confirm the absence of sustained contractions to UTP and UDP in P2Y_6_‐R KO mice (Haanes et al., 2016; Kauffenstein et al., 2016), but, in contrast, we cannot confirm that MT is abolished. Any functional effect in a knockout or transgenic mouse strain may be affected by bias or loss of redundant pathways. T‐type Ca^2+^ channels are involved in MT development at lower pressures, and L‐type Ca^2+^ channels dominate MT at higher pressures (Björling et al., 2013). It is tempting to speculate that the P2Y_6_‐R KO mice in that study (Kauffenstein et al., 2016) had reduced T‐type and L‐type channel expression, explaining the loss of MT. In contrast, the P2Y_6_‐R WT might have had reduced L‐type channel expression in mesenteric arteries, explaining the unusual bell‐shaped MT pressure curve. In the present study, we ruled out a role for the endothelium or upregulation of P2Y_2_‐Rs to compensate for the deletion of vascular smooth muscle cell (VSMC) P2Y_6_‐Rs in the P2Y_6_‐R KO mice. Since KCl‐ and noradrenaline‐induced constriction were also normal, we argue that we have ruled out plausible bias or compensatory regulation that could explain why we do not observe any role of P2Y_6_‐Rs in MT.

Prompted by previous studies showing an involvement of SK and S1P‐receptors in MT (Bolz et al., 2003; Hui et al., 2015), and by the abundant expression of S1pr2 (S1P_2_‐Rin mouse mesenteric arteries (present study), we tested the effects of inhibition of SK and S1P_2_‐R using the compounds SKI‐II and JTE‐013, respectively. The SK inhibitor robustly inhibited MT at high pressures (100–120 mmHg) without affecting resting diameter or KCl‐induced vasoconstriction at low pressure (40 mmHg). We thus inferred that the effect of SKI‐II was significant and selective for sphingosine kinase and therefore tested S1P_2_‐R inhibitor JTE‐013 at two concentrations (5 vs. 1.6 µM) that did not block the KCl‐induced constrictions. MT was nearly abolished by JTE‐013 in both young and middle‐aged mice, showing that S1P_2_‐R could be the pressure‐sensing mechanism in MT development, or could be responding to local release of S1P derived from pressure‐dependent activation of SK. The lack of effect of JTE‐013 on Ca^2+^ signalling in cultured human coronary artery SMCs and no effects of an S1P_3_‐R blocker on MT clearly argue that the JTE‐013‐induced inhibition of MT is specific for S1P_2_‐R. The absolute magnitude of the inhibition of MT by JTE‐013 was larger in young mice compared to middle‐aged mice. The same was true for the effect in WT littermate versus transgenic 5xFAD mice carrying mutations for familial Alzheimer's disease. The reduction in baseline MT in both middle‐aged and 5xFAD‐TG mice precludes conclusions about a differential role of S1P_2_‐R in ageing or 5xFAD pathology. Instead, we propose that reduced MT in 5xFAD mice may result from blood–brain barrier leakage of β‐amyloid and inflammatory cytokines into the systemic circulation, inducing low‐grade chronic inflammation in mesenteric arteries (Cifuentes et al., 2025; Ronnback & Hansson, 2019; Storck et al., 2016). Conversely, it can be hypothesised that systemic factors associated with AD pathology may contribute to the observed reduction in MT in 5xFAD mice. Cerebral blood flow autoregulation is impaired in mice overexpressing amyloid precursor protein (APP) (Niwa et al., 2002), and it has been documented that peripheral accumulation of Aβ_1‐42_ proteins can impair cardiac function (Huang et al., 2025; Murphy et al., 2022). Whether these mechanisms also play a role in the impaired function of systemic arteries remains to be established. The question whether the pressure‐sensing mechanism in small arteries involves a local factor (autocoid) released from within the vascular wall or is reliant on mechanical activation of a cellular signalling event deserves attention. Our data indicated that the endothelium did not play a role in MT development, ruling out actions of autocoids released from endothelial cells. Moreover, only partial inhibition of MT resulted from blocking the production of S1P via SK, whereas near abolishment of MT was observed by blocking the S1P_2_‐R. Thus, our data suggest a partial role of local S1P production in VSMCs, and partial mechanical stimulation of S1P_2_‐R or a parallel unknown mechanism involved in S1P production.

A recent review found that in a majority (13 of 15) of animal studies across several vascular beds that the myogenic tone is reduced by ageing (Jensen, 2024). In relation to the present study, the myogenic tone in small mesenteric arteries was unequivocally found to be reduced in middle‐aged and old rats and mice. However, in rat cerebral arteries and in mouse parenchymal arterioles, the endothelium seemed to play a modulatory role supporting myogenic tone at old age, such that only endothelium‐denuded preparations showed a reduced myogenic tone in ageing, like other vascular beds (Geary & Buchholz, 2003; Polk et al., 2023). In mice, the myogenic tone was also reduced by ageing in middle cerebral arteries (Springo et al., 2015; Toth et al., 2013a, 2013b). Taken together, most evidence points to an impairment of myogenic tone in ageing, except for some conditions in which the cerebral endothelium may release a vasoconstricting factor in ageing (Jensen, 2024). Our previous proteomic study of the effects of ageing in small cerebral and mesenteric arteries from mice concluded that regulation of the actin cytoskeleton and the RhoA/ROCK pathway was involved in the age‐dependent vascular dysfunction (Rabaglino et al., 2021). To further investigate the possible role of ROCK in age‐dependent vascular dysfunction, we performed in vivo blocker experiments using SRM to measure resting diameters in cerebral arteries and arterioles in anaesthetised young and middle‐aged mice. The SRM measurements showed that blockade of S1P_2_‐R and ROCK2 predominantly dilated the smaller arteries and arterioles, in contrast to the effect of L‐type channel inhibition, which had similar efficacy in small and large arteries. However, there were no age‐dependent differences in the effects of the three blockers in either small or large cerebral arteries. Whilst the calibre‐dependent effects of S1P_2_‐R and ROCK inhibition agree with a role in setting the resting diameter in resistance arteries, the data do not directly support a role for S1P_2_‐R or ROCK2 in age‐dependent vascular dysfunction. Based on our previous proteomic study (Rabaglino et al., 2021), a significant down‐regulation or age‐dependent interaction of proteins regulating the activities of the VSMC contractile mechanism (such as ROCK, Myosin Light Chain Phosphatase or Myosin Light Chain) is still our suggested mechanism to explain the impaired MT in middle‐aged mice. Future studies must shed more light on the specific roles of individual proteins in this age‐dependent process.

A model for the proposed role of SK and S1P_2_‐R in myogenic tone development, via activation of RhoA/ROCK pathway and calcium sensitisation of VSMCs, is shown in Figure 13. To conclude, it is highly likely that local sphingosine kinase‐dependent release of S1P from the vascular wall and activation of S1P_2_‐receptors play an important role in the pressure‐sensing step in myogenic tone development in small arteries.

## AUTHOR CONTRIBUTIONS

*The ex vivo experiments (pressure myography, qPCR, calcium imaging) were performed in the laboratory of the University of Copenhagen*: Lars Jørn Jensen. *The in vivo Synchrotron Radiation Microangiography experiments were prepared in the laboratory of the National Cerebral and Cardiovascular Center Research Institute (Osaka, Japan) and performed at the SPring‐8 large synchrotron radiation facility in Hyogo prefecture, Japan*: James Todd Pearson. *Contributed to the conception or design of the work*: Gry Freja Skovsted, James Todd Pearson and Lars Jørn Jensen.

All authors contributed to the acquisition, analysis or interpretation of data for the work. In addition, all authors contributed to drafting the work or revising it critically for important intellectual content. Finally, all authors approved the final version of the manuscript and agree to be accountable for all aspects of the work. All individuals designated as authors qualify for authorship, and all those who qualify for authorship are listed.

## CONFLICT OF INTEREST

The authors declare no conflict of interest.

## Data Availability

All data supporting the results in the paper are in the paper itself, and no shared data were used.
